# Functional Evaluation of Genetic and Environmental Regulators of P450 mRNA Levels

**DOI:** 10.1371/journal.pone.0024900

**Published:** 2011-10-05

**Authors:** Dazhi Wang, Zhengwen Jiang, Zhongyang Shen, Hui Wang, Beilan Wang, Weihua Shou, Hong Zheng, Xun Chu, Jinxiu Shi, Wei Huang

**Affiliations:** 1 Rui Jin Hospital, School of Medicine, Shanghai Jiao Tong University, Shanghai, China; 2 Department of Genetics, Shanghai-MOST Key Laboratory of Health and Disease Genomics, Chinese National Human Genome Center, Shanghai, China; 3 Organ Transplant Center, Tianjin First Central Hospital, Tianjin, China; Institut Jacques Monod, France

## Abstract

Variations in the activities of Cytochrome P450s are one of the major factors responsible for inter-individual differences in drug clearance rates, which may cause serious toxicity or inefficacy of therapeutic drugs. Various mRNA level is one of the key factors for different activity of the major *P450* genes. Although both genetic and environmental regulators of *P450* gene expression have been widely investigated, few studies have evaluated the functional importance of cis- and trans-regulatory factors and environmental factors in the modulation of inter-individual expression variations of the *P450* genes. In this study, we measured the mRNA levels of seven major *P450* genes (*CYP1A1*, *CYP1A2*, *CYP2C9*, *CYP2C19*, *CYP2D6*, *CYP3A4* and *CYP3A5*) in 96 liver biopsy samples from Chinese population. Both trans-acting (mRNA levels and non-synonymous SNPs of putative regulator genes) and cis-acting (gene copy number and functional SNPs) factors were investigated to identify the determinants of the expression variations of these seven *P450* genes. We found that expression variations of most *P450* genes, regulator genes and housekeeping genes were positively correlated at the mRNA level. After partial correlation analysis using *ACTB* and *GAPDH* expression to eliminate the effect of global regulators, a UPGMA (Unweighted Pair Group Method with Arithmetic Mean) tree was constructed to reveal the effects of specific regulation networks potentially masked by global regulators. Combined with the functional analysis of regulators, our results suggested that expression variation at the mRNA level was mediated by several factors in a gene-specific manner. Cis-acting genetic variants might play key roles in the expression variation of *CYP2D6* and *CYP3A5*, environmental inducers might play key roles in *CYP1A1* and *CYP1A2* variation and global regulators might play key roles in *CYP2C9* variation. In addition, the functions of regulators that play less important roles in controlling expression variation for each *P450* gene were determined.

## Introduction

The Cytochrome P450 superfamily is a very large and diverse group of hemoproteins that is present in almost all living organisms. About 57 putative functional Cytochrome *P450* genes and more than 58 pseudogenes have been identified in the human genome [1], [2]. These *P450* genes encode enzymes, such as monooxygenase, that play a crucial role in detoxification of exogenous xenobiotics, decomposition of drugs and metabolism of many endogenous compounds, such as hormones, fatty acids, prostaglandin, cholesterol and vitamin D [3], [4]. It is well recognized that inter-individual variability in activity of the P450 enzymes is a major factor responsible for inter-individual variations in drug clearance rates [5]. Cytochrome P450 enzymes, which play key roles in Phase I oxidative metabolism, have been extensively investigated due to their great variations in activity in the human population. The inter-individual variability in the total activity of P450s is primarily caused by polymorphisms that affect activity and expression. Strong correlations between expression level and enzyme activity have been observed for *CYP1A1*, *CYP1A2*, *CYP3A4*, *CYP2C8*, *CYP2C9*, *CYP2D6* and *CYP2B6*, suggesting that the mRNA level may be a major determinant of the total activity of these *P450* genes [6], [7].

Ten- to a hundred-fold inter-individual differences in expression levels have been observed for most Cytochrome *P450* genes [6], [8]. Most of the P450s that metabolize exogenous compounds are highly expressed in the human liver, but some P450 forms are expressed at low levels in extrahepatic tissues. The mechanisms underlying the maintenance and regulation of the high expression levels of the *P450* genes in the liver are not completely understood. Data have shown that the expression levels of some liver-enriched transcription factors, such as the Constitutive Androstane Receptor (CAR), Hepatic Nuclear Factor 4α (HNF4α) and P450 Oxidoreductase (POR), may determine the variability in the basal expression and activity of a broad range of *P450* genes [8]. In addition, *P450* gene-specific mechanisms have been identified. For example, activation of the aryl hydrocarbon receptor (AHR)-mediated pathway induces an increase in expression of the *CYP1A1* and *CYP1A2* genes [9], [10]; for *CYP2D6* and *CYP2A6*, copy number variation and some regulatory alleles have been reported to be important for expression regulation [11], [12]. A polymorphism in CYP3A5, the CYP3A5*3 allele, is the major factor that modulates expression [13]. Despite the tremendous amount of research that has been performed to construct the regulatory network of *P450* genes, few studies have evaluated the functional importance of various factors in controlling *P450* expression variation among individuals.

In this study, we systematically evaluated the determinants of *P450* expression variation among individuals. We measured the absolute expression levels of three housekeeping genes (*18S rRNA*, *GAPDH* and *ACTB*), seven *P450* genes (*CYP1A1*, *CYP1A2*, *CYP2C9*, *CYP2C19*, *CYP2D6*, *CYP3A4* and *CYP3A5*) and seven putative Cytochrome P450 regulator genes (*USF1*, *CAR*, *PXR*, *HNF4A*, *HNF1A*, *AHR* and *ARNT*) (for full names of these genes, refer to Table S1). Pairwise correlation analysis was performed to explore the network of interaction among these genes. The gene-gene interactions and gene-environment interactions made it difficult to distinguish the effects of each regulatory factor. Therefore, allelic expression ratios (AERs) of *CYP1A1*, *CYP1A2*, *CYP2C9*, *CYP2C19* and *CYP3A4* were measured using two SNP markers from each gene to determine the presence of cis-acting regulatory variants [14]–[17]. The copy number of *CYP2D6* was determined, and the correlation between the copy number and mRNA level of *CYP2D6* was examined. Fifteen SNPs located in the seven *P450* genes and three regulator genes (Table S2) were typed and tested for association with the expression levels of the corresponding *P450* genes.

## Results

### Expression variations in the *P450*s and regulatory genes

**Figure 1:**
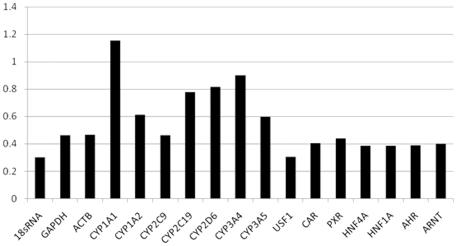
Coefficients of variation of transcript molecule numbers in 96 Chinese liver biopsy samples for 17 genes tested.

**Figure 2:**
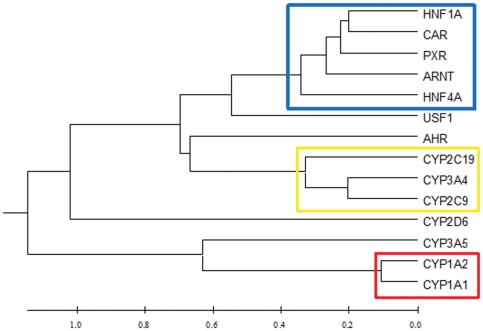
A UPGMA tree showing the relationships between 14 genes based on expression levels. Spearman's correlation coefficients between the mRNA levels of seven *P450s* and seven regulator genes were identified using SPSS15.0. A distance matrix was constructed from the negative natural logarithm of the absolute value of the correlation coefficient, and the UPGMA tree was constructed from this distance matrix with MEGA 4.0. Three clusters were well defined: one containing CYP1A1 and CYP1A2; one containing CYP2C9, CYP2C19 and CYP3A4 and one containing HNF4A, HNF1A, ARNT, PXR and CAR.

The mRNA levels of two putative housekeeping genes (*GAPDH* and *ACTB*), seven *P450* genes (*CYP1A1*, *CYP1A2*, *CYP2C9*, *CYP2C19*, *CYP2D6*, *CYP3A4* and *CYP3A5*) and seven regulatory genes (*USF1*, *CAR*, *PXR*, *HNF1A*, *HNF4A*, *AHR* and *ARNT*) were determined by quantitative real-time PCR and normalized to *18S rRNA* expression. Up to 8- to 10-fold inter-individual differences were observed for *GAPDH* and *ACTB* (Table S4). This result raised doubts about the applicability of *GAPDH* and *ACTB* as reference genes. Thus, *18S rRNA* was selected as the reference gene based on two observations: 1) the *18S rRNA* level had the lowest CV(coefficient of variation) as shown in Fig. 1 and 2) the *18S rRNA* level is strongly correlated (r = 0.76) with the cDNA level of *USF1*, which had the second lowest CV and encodes a ubiquitous transcription factor.

In our samples, all regulatory genes were constitutively transcribed at low levels with low inter-individual variability (<7-fold), whereas most *P450* genes showed much greater inter-individual variability (>13-fold), except for *CYP2C9* (7.2-fold). The expression levels of the seven *P450* genes were *CYP3A4*>*CYP2C9*>*CYP2D6*>*CYP1A2*>*CYP3A5*>*CYP2C19*>*CYP1A1* (Table S4). The expression variations were *CYP1A1*>*CYP2D6*≈Y*CYP3A4*>*CYP2C19*>*CYP3A5*≈*CYP1A2*>*CYP2C9* (Table S4). One sample with no *CYP2D6* expression was excluded from the variation comparison due to the loss of both *CYP2D6* alleles (as discussed later). In some individuals, extreme differences in expression levels were observed for *CYP3A4*, *CYP2D6* and *CYP1A1* by comparison of the T5/B5 and Max/Min ratios. The T5/B5 ratio denotes the ratio of the average expression level of the top 5% of samples to that of the bottom 5% of samples. There was more than a 5-fold difference between these two ratios for *CYP3A4* (Table S4).

### Correlations among mRNA levels of *P450* and regulator genes

Interestingly, the expression levels of almost all of the genes (including *GAPDH* and *ACTB*) were strongly correlated with each other at the mRNA level (Table S5). Such correlations may be the result of powerful global regulators. Thus, we conducted a partial correlation analysis using the mRNA levels of both *GAPDH* and *ACTB* to eliminate or reduce these effects. After the partial correlation analysis, the degree of most correlations was largely reduced, and many were not significant (p>0.05) (Table S6). However, strong correlations were still observed between *CYP1A1* and *CYP1A2* (ρ = 0.81); *CYP2C9* and *CAR* (ρ = 0.66), *PXR* (ρ = 0.57), *HNF1A* (ρ = 0.50) and *ARNT* (ρ = 0.57); among *ARNT*, *HNF1A*, *HNF4A*, *PXR* and *CAR* (ρ>0.44) and among *CYP2C9*, *CYP2C19* and *CYP3A4* (ρ>0.44).These correlations remained significant after adjustment for age and smoking history in multivariate logistic regression analysis (p_adj_<0.001). A UPGMA (Unweighted Pair Group Method with Arithmetic Mean) tree (Fig. 2) was constructed based on the partial correlation matrix, and three clusters were roughly defined. The first cluster was composed of five regulator genes (*HNF1A*, *HNF4A*, *CAR*, *PXR* and *ARNT*), the second contained *CYP2C9*, *CYP3A4* and *CYP2C19* and the third included only *CYP1A1* and *CYP1A2*.

### Copy number variation and expression level of *CYP2D6*

**Figure 3:**
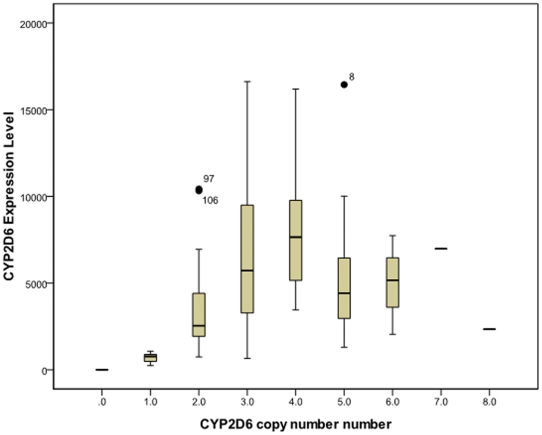
Relationship between copy number and the mRNA level of CYP2D6. Both deletion and multiple duplications of CYP2D6 were observed in the 96 samples. One, seven and fourteen samples were identified as 0, 1 and ≧5 copies of CYP2D6, respectively. Increasing copy numbers tended to increase the expression level for samples with <5 copies but not for those with ≧5 copies.

The copy number of *CYP2D6* varied from 0 to 9 among the 92 DNA samples (Fig. 3). Four samples were excluded due to inconsistency between measurements using different *CYP2D6* primers or without mRNA. One sample containing a homozygous *CYP2D6* deletion (CYP2D6*5) was identified. The mRNA level of *CYP2D6* is plotted against copy number in Fig. 3. Higher copy numbers tended to increase the expression level in samples with ≤4 copies. A strong correlation (ρ = 0.63, p<0.0001) was observed between copy number and expression level for samples with no more than four copies of *CYP2D6*. The correlation remained significant after adjustment for age and smoking history in multivariate logistic regression analysis (p_adj_<0.001).

### Association analysis between genetic variants in *P450s* and regulator genes and mRNA levels of seven *P450* genes

**Figure 4:**
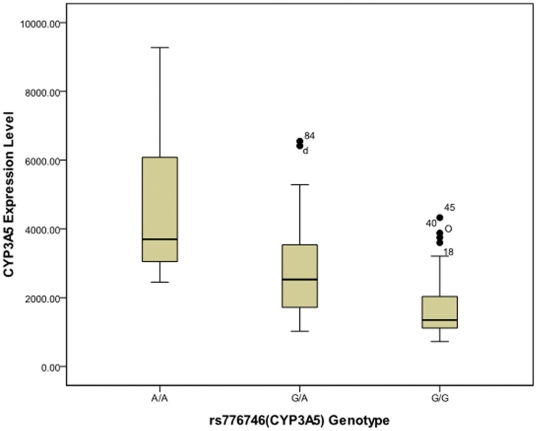
Correlation between genotypes at rs776746 and the expression level of CYP3A5. A is the wild-type allele and G is the mutant allele (CYP3A5*3), which results in aberrant splicing. The natural logarithm of the mRNA data was used for ANOVA analysis. ρ denotes the Spearman's correlation coefficient. The Boxplot displays the minimal, lower quartile, median, upper quartile, maximal points and outliers (marked by circle and sample No.).

Expression variations in the *P450* genes may result from the different activities of regulator protein isoforms in addition to different protein levels. All three non-synonymous SNPs in the regulatory genes mentioned above with MAF (minor allele frequency)>5% in the Chinese population were genotyped in 96 liver samples. These three SNPs, two (rs1169288 and rs2464196) in *HNF1A* and one (rs2066853) in *AHR*, were not associated with the expression levels of any of the seven *P450* genes examined, which indicates that the polymorphisms of HNF1A and AHR do not contribute to the expression variations of these seven *P450* genes. In total, 12 marker SNPs in the *P450* genes (Table S2) used for allelic expression imbalance (AEI) detection were tested for association with mRNA levels. Rs776746 (also known as 6986G>A) in *CYP3A*5, an A>G substitution in intron 3 that causes aberrant splicing, was strongly correlated with the mRNA level of *CYP3A5* (ρ = 0.506, Fig. 4). The correlations remained significant after adjustment for age and smoking history in multivariate logistic regression analysis (p_adj_<0.001). Marginal associations were observed between rs17861162 and the *CYP1A2* mRNA level (p = 0.044); rs1934967 and the *CYP2C9* mRNA level (p = 0.025) and rs33972239 and the *CYP3A4* mRNA level (p = 0.036) by the Kruskal-Wallis test but not by ANOVA. There was also a marginal association between rs4244285 and the *CYP2C19* mRNA level (p = 0.047) detected by ANOVA but not by the Kruskal-Wallis test. These associations were no longer significant after adjustment for age and smoking history in multivariate logistic regression analysis (p_adj_>0.05).

## Discussion

### Expression variations of P450s and regulator genes

*CYP1A1* showed the largest inter-individual difference in expression (138-fold, Table S4), which is consistent with the observation that expression of *CYP1A1* was highly inducible by environmental inducers. The levels of inter-individual expression variability observed in this study for most of the *P450* genes and some regulatory genes were much lower than those found in several previous studies [6], [8]. For example, an approximately 6-fold difference was detected in our samples for *CAR*, but Wortham et al. [8] reported a 145-fold difference. This study found differences of approximately 14- and 64-fold for *CYP1A2* and *CYP3A4*, respectively, whereas differences of 582- to 3638-fold were reported for *CYP1A2* and differences of 860- to 1281-fold were reported for *CYP3A4* by Wortham et al. [8] and Rodríguez-Antona et al. [6], respectively. Some studies have found levels of variability similar to our results, such as differences of 118-fold and approximately 15-fold for *CYP3A4* [2] and *CYP1A2* [18], respectively.

The inconsistency among reports may be attributed to differences in ethnic groups, environmental factors, RNA quality and experiment design. In our study, the samples were obtained from Chinese Han healthy liver donors and treated with the protocols recommended by Qiagen. Most primers were designed in a region close to the 3′ end of the mRNA to amplify a short fragment of 60–180 bp in length, which should reduce the variation resulting from low RNA quality. Moreover, all SNPs in the sequences used for primer design were masked first by performing a BLAST search of the SNP database; therefore, the primers did not harbor any SNP, which prevented differences in amplification efficacies due to cDNA samples with different genotypes at SNPs located within primer sequences.

In our study, measures were taken to reduce the noise effects of non-genetic factors. Besides the genetic factors, the inter-individual mRNA expression variations of P450 may be due to differences in gender, age, food intake, drug treatment, smoking, alcohol using etc. All the subjects recruited in this study were males and between 30 to 45 years old. According to the donors' statements, no drugs were taken before the surgeries for at least 30 days. Alcohol using and smoking were prohibited 30 days prior to surgery, which avoided the confounding by alcohol using and smoking. Multivariate logistic regression was used to adjust for potential confounding factors including age and smoking history.

However, differences in the food intake of the 96 participants were not considered, which might be a resource of bias in our study. Additionally, the expression variation caused by rare alleles may not be evident in our limited sample size. Thus, further studies are needed to demonstrate the expression variations of the *P450*s in larger sample sizes to eliminate bias.

### Coexpression analysis

Unexpectedly, strong correlations were detected among almost all genes examined in this study, including *GAPDH* and *ACTB*, which were not thought to be involved in the *P450* regulation network (Table S5). Yang et al. [7] suggested that liver P450s are situated at the center of many endocrine and xenobiotic metabolic pathways, which require cross talk among numerous nuclear receptor networks. Alternatively, it is also reasonable to assume that this correlation may be caused by powerful global regulatory factors that control expression of most active genes, which would mask the true regulation network of *P450*s and the corresponding regulator genes. Such global regulatory factors are most likely basic transcription factors and ubiquitous chromatin modifiers. We performed a partial correlation analysis using the expression levels of *GAPDH* and *ACTB* as controls for the elimination of the presumed effect. Not surprisingly, 39% of the correlations were eliminated (Table S6). Based on the remaining “corrected” correlations, we performed a cluster analysis and constructed the UPGMA tree with MEGA 4 [19]. Finally, three clusters were roughly identified (Fig. 2).

In cluster I, strong correlations remained among *CAR*, *PXR*, *HNF1A*, *HNF4A* and *ARNT* (Fig. 2), which suggested that there was co-regulation or interaction among these genes. *HNF1A*, which is regulated by *HNF4A* [20], [21], displayed the three strongest correlations with *CAR* (ρ = 0.67), *PXR* (ρ = 0.66) and *ARNT* (ρ = 0.63), indicating that *HNF1A* may be important for regulating the expression of *CAR*, *PXR* and *ARNT*. Of all of the correlations between *HNF4A* and other regulator genes, the correlation between *HNF4A* and *HNF1A* was the strongest (ρ = 0.545), which is consistent with the observation that *HNF1A* is regulated by *HNF4A*. Regulation of *PXR* by *HNF1A* is supported by a previous report that a putative *HNF1A* binding site is present in the human *PXR* promoter and a 6-bp deletion at this site diminished the activity of hPAR-2, one of the *PXR* transcripts [22]. However, limited data have uncovered a relationship between *HNF1A* and *CAR* or *ARNT*. Here, we propose a simplified regulation network among *CAR*, *PXR*, *HNF1A*, *HNF4A* and *ARNT*, in which *HNF4A* regulates the expression of *HNF1A*, and *HNF1A* further regulates the expression of *CAR*, *PXR* and *ARNT*. Other interactions among *HNF4A*, *ARNT*, *CAR* and *PXR* are possible, and more data must be collected to evaluate this hypothesis. Clusters II and III showed co-expression of the *P450* genes. Because *P450s* are evolved from a common ancestor gene, the correlations among the *P450* genes [1], [7], [23] might result from the shared regulatory factors. *CYP1A1* and *CYP1A2* in cluster III are orientated in a head-to-head configuration on 15q22-q24. The 23.3 kb intergenic region might harbor certain element with a role in co-regulation of the expression of *CYP1A1* and *CYP1A2* [24]. However, the correlation did not entirely agree with the sequence similarity [1]. The effects of specific regulatory factors on each *P450* gene are discussed in detail below.

### *CYP1A1* and *CYP1A2*

**Figure 5:**
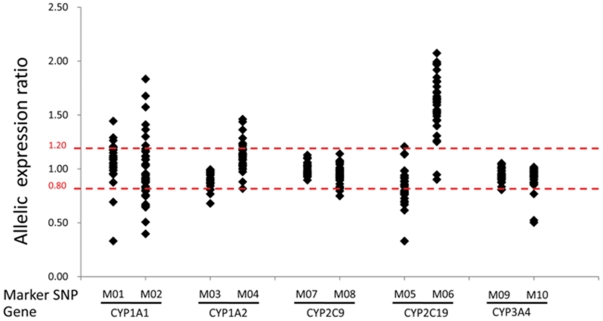
Distribution of allelic expression ratios of five *P450* genes. M01 to M10 denotes markers SNP 1 to 10, which are rs1048943, rs4646421, rs17861162, rs762551, rs4388808, rs4244285, rs1934967, rs1934969, rs2246709 and rs33972239, respectively, in CYP1A1, CYP1A2, CYP2C19, CYP2C9 and CYP3A4. The marker SNP is either intronic or exonic, and the ratio of two alleles of marker SNP in the cDNA is representative of the allelic expression ratio of the corresponding gene. The upper and lower dashed lines indicate AERs of 1.2 and 0.8, respectively. In general, an AER of less than 0.8 or greater than 1.2 is experientially indicative of allelic expression imbalance.

*CYP1A2* is constitutively expressed in the liver. *CYP1A2* and *CYP1A1* are induced by various types of environmental chemicals, such as PAHs (polycyclic aromatic hydrocarbons), HAAs (heterocyclic aromatic amines amides) and HAHs (halogenated aromatic hydrocarbons) via the ligand-activated AHR-ARNT pathway. A strong correlation (ρ = 0.81) between the expression levels of *CYP1A1* and *CYP1A2* demonstrated that the two *CYP1A* genes are co-regulated. However, the mRNA levels of *CYP1A1* and *CYP1A2* were not correlated with those of most of the regulator genes, except for the weak correlations observed between *CYP1A1* and *USF1* (p = 0.037) and between *CYP1A2* and *AHR* (p = 0.025), *CAR* (p = 0.024) and *USF1* (p = 0.009), which indicate that environmental inducers involved in activation of the AHR-ARNT pathway may be an important determinant for the variability in mRNA levels of *CYP1A1* and *CYP1A2*. Quantitation of the active and total AHR protein levels may provide direct evidence to support this hypothesis. Cis-acting variants may also account for the variations, albeit it to a small extent, as described in Fig. 5. These cis-acting variants were estimated to induce inter-individual expression differences of only <2.5-fold for *CYP1A1* and <1.7-fold for *CYP1A2*, compared with a total variability in mRNA level of 138-fold for *CYP1A1* and 14-fold for *CYP1A2*. After exclusion of four outlier samples for the mRNA levels of *CYP1A2*, the inter-individual expression difference was about 9-fold, and thus the cis-acting genetic variants may account for up to 20% of the inter-individual variability. Therefore, the major determinants for variability in the expression levels of *CYP1A1* and *CYP1A2* may include environmental factors involved in activation of the AHR-ARNT pathway, global regulators as described above and, to a lesser extent, cis-acting variants.

### *CYP2C9*

CYP2C9 is the most abundant CYP2C subfamily isozyme in human liver [25]–[27]. Expression of CYP2C9 has been reported to be regulated by a wide range of liver-enriched transcription factors, such as CAR, PXR and HNF4A [28]. Significant correlations between the mRNA levels of *CYP2C9* and all seven regulator genes, especially *CAR*, *PXR*, *ARNT* and *HNF1A* (ρ>0.5), were observed, which provides evidence that expression of putative *P450* regulator genes contributes to the inter-individual variability of *CYP2C9* expression levels. Although HNF4A has been reported to regulate *CYP2C9* expression, our results suggest that HNF1A may play a more important role in the regulation of *CYP2C9* expression than HNF4A because a stronger correlation was observed between the mRNA levels of *CYP2C9* and *HNF1A* than between those of *CYP2C9* and *HNF4A* (Table S4). In addition, our study shows for the first time that ARNT may be an important regulator of *CYP2C9*. Strong correlations among *CYP2C9*, *CYP2C19* and *CYP3A4* support the reports that these three genes are co-regulated by CAR and PXR [29]. No significant common cis-acting genetic variants were detected for *CYP2C9* (Fig. 5), which is consistent with previously reported data [17]. In conclusion, of the seven *P450* genes examined, *CYP2C9* displayed the least expression variation in 96 Chinese liver biopsy samples, and the variability in expression was mainly determined by expression of *P450* regulator genes, such as CAR, PAR, HNF1A and ARNT, in addition to the global regulators as described above. Environmental inducers may also account for some variation in *CYP2C9* expression by activating CAR or PXR.

### *CYP2C19*

Up to 45-fold inter-individual differences were detected for *CYP2C19* among the 96 Chinese liver samples. Only low correlations between *CYP2C19* and several regulator genes (*PXR*, *CAR*, *ARNT* and *HNF1A*) were observed. The genetic variant CYP2C19*2 at rs4244285, which results in aberrant splicing, may account for up to 2-fold allelic expression difference (Fig. 5); however, only a weak association was detected between rs4233285 and the mRNA levels of *CYP2C19* by ANOVA. Therefore, the inter-individual variability in the mRNA levels of *CYP2C19* (up to 45-fold) may be largely determined by factors other than cis-acting genetic variants or the mRNA levels of regulator genes.

*CYP2C19* is highly induced by drugs, such as rifampin, via activation of the CAR/PXR-mediated pathway [29]. Strong correlations were found among the mRNA levels of three target genes of CAR/PXR: *CYP2C19*, *CYP2C9* and *CYP3A4*. However, slight correlations between the mRNA levels of *CYP2C19* and *CAR* and *PXR* suggest the importance of CAR/PXR activation by *CYP2C19* inducers in modulating the expression of *CYP2C19*. Quantitation of the active and total CAR or PXR protein levels may provide further evidence for this hypothesis. Therefore, the major determinants for the inter-individual variability in the mRNA levels of *CYP2C19* may include global regulators and environmental inducers for activation of the CAR/PXR pathway. To a lesser extent, cis-acting genetic variants or the mRNA levels of *CAR* and *PXR* may account for a small part of the expression variation of *CYP2C19*.

### *CYP2D6*

A substantial inter-individual difference was identified (up to 68-fold without inclusion of a sample homozygous for *CYP2D6* deletion) at the mRNA level for *CYP2D6*. Slight correlations were observed between *CYP2D6* and several regulator genes (*PXR*, *CAR*, *HNF4A* and *ARNT*) at the mRNA level, demonstrating that the mRNA levels of regulator genes contribute to the variability in expression of *CYP2D6* (Table S6), albeit to a much lesser extent compared with *CYP2C9*. The global factor that affected expression of most of the other genes played a less important role in *CYP2D6* expression (Table S6). The copy number of functional *CYP2D6* was the major determinant of the variability in the mRNA levels of *CYP2D6* (Fig. 3). The *P450* regulator genes and global regulators may make some contributions but to lesser extents.

The correlation between the copy number and mRNA level of *CYP2D6* was not consistent when the copy number is more than four in this study. The inconsistency may attribute to two reasons. First, the inconsistency may be caused by the insufficient sample size. Thirteen samples with more than four copies of *CYP2D6* were investigated in current study. Even larger sample size is needed in the further study to validate whether this inconsistency is true. Second, the *CYP2D6* gene duplications include functional, partly functional and nonfunctional genes, which could cause splicing defeat or produce unstable mRNA [30]–[32]. It is possible that the samples in our study with more than four *CYP2D6* copies contain partly functional or nonfunctional gene copies, which results in this inconsistency. Further long range PCR and clone sequencing are needed to clarify this issue.

### *CYP3A4*

High expression levels and high inter-individual expression differences were observed in *CYP3A4*. The slight correlations observed between *CYP3A4* and several regulator genes (*PXR*, *CAR*, *HNF1A* and *ARNT*) at the mRNA level indicated that regulator genes have moderate effects on *CYP3A4* expression variation (Table S6). CYP3A4 is highly induced by drugs, such as rifampin, via activation of the CAR/PXR-mediated pathway. Similarly to *CYP2C19*, the determinants for the expression variability of *CYP3A4* may be global regulators, environmental inducers that activate CAR/PXR and, to a lesser extent, the mRNA levels of the *CAR* and *PXR* genes. Some rare cis-acting genetic variants might induce up to 2-fold inter-individual expression differences; however, only a very small proportion of samples (4%) was affected (Fig. 5). Thus, these genetic variants contribute little to the variability in the mRNA levels of *CYP3A4*. In summary, the determinants for the expression variability of *CYP3A4* may be global regulators, environmental inducers that activate CAR/PXR and, to a lesser extent, the mRNA levels of the *CAR* and *PXR* genes.

### *CYP3A5*

No correlations were observed between the mRNA levels of *CYP3A5* and the regulatory genes. A strong association (p<0.0001, Fig. 4) was identified between the mRNA levels of *CYP3A5* and rs776746, which is the CYP3A5*3 allele that induces aberrant splicing of *CYP3A5*. The unstable mRNA produced by aberrant splicing decreases the expression level. Thus, this cis-acting element is another determinant of CYP3A5 expression variation in addition to global regulators.

Based on the data reported here, we propose that at least four factors are determinants of the expression variation of *P450* genes (Table 1): I) global regulators that affect the expression levels of most active genes, which are most likely basic transcription factors and ubiquitous chromatin modifiers; II) *P450*-specific regulators; III) environmental inducers responsible for the activation of *P450*-specific regulators and IV) genetic variants of the *P450* genes, including gene duplication or deletion. In our study, we weighed the functional importance of each factor on the expression variation of each *P450* gene.

**Table 1:** Contributions of four major determinants to the variability in mRNA levels of P450 genes.

	Global Regulators^1^	P450-specific Regulators^2^	Environmental Factors^3^	Cis-acting Genetic Variants^4^
*CYP1A1*	**	NS	****	*
*CYP1A2*	***	*	****	*
*CYP2C9*	****	***	***	NS
*CYP2C19*	**	**	***	*
*CYP2D6*	*	**	NS	***
*CYP3A4*	***	**	***	NS
*CYP3A5*	***	NS	NS	***

NS denotes not significant. The number of asterisks (*) corresponds to the degree of contribution by different determinants.

1 : The number of asterisks (*) was determined based on the correlation coefficients between the mRNA level of GAPDH and that of each P450 gene (one asterisk for a rho score of approximately 0.2).

2 : The number of asterisks (*) was determined based on the correlation coefficients between the mRNA levels of P450 regulator genes and that of each P450 gene (one asterisk for a rho score of approximately 0.2).

3 : The number of asterisks (*) was determined by the correlation coefficients between the mRNA levels of P450 genes under coregulation (one asterisk for a rho score of approximately 0.2).

4 : The number of asterisks (*) was arbitrarily determined based on the allelic expression ratios (for *CYP1A1*, *CYP1A2* and *CYP2C19*) or the correlation coefficients between the mRNA level and copy number or some SNPs (for *CYP2D6* and *CYP3A5*).

In summary, we functionally evaluated several factors that control the variability in the mRNA levels of *CYP1A1*, *CYP1A2*, *CYP2C9*, *CYP2C19*, *CYP2D6*, *CYP3A4* and *CYP3A5*. Moreover, the variations in the mRNA levels of 7 *P450* genes characterized in 96 Chinese liver samples in this study provide useful data on the liver *P450*s in Asian populations, which traditionally have not been analyzed in European-based studies.

Although strong correlations were observed between the mRNA level and certain variants (*CYP3A5* and rs776746; *CYP2D6* and the copy number), such genetic variation is not good enough to be used clinically in the individual patient. The activities of drug-metabolizing enzymes are regulated at many levels except for genetic factors. Further studies should be done before the application of the research conclusions in clinical practice.

## Materials and Methods

### Ethics Statement

All participants gave written informed consent. The acquisition of all data using samples from these participants was approved by the Ethics Review Committee of the Chinese National Human Genome Center at Shanghai.

### Human Liver Tissues

A total of 96 human liver biopsy samples were obtained in Tianjin First Center Hospital from donor livers that were morphologically normal and tested negative for HIV (human immunodeficiency virus) and hepatitis. All the subjects recruited in this study were males and between 30 to 45 years old. According to the donors' statements, no drugs were taken before the surgeries for at least 30 days. Alcohol using and smoking were prohibited 30 days prior to surgery. About 50–200 mg of tissue for each sample was immediately submerged in RNAlater (Sigma-Aldrich) after collection from live livers during liver transplant surgery. After storage overnight in RNAlater at 4°C, all samples were stored at −70°C until the DNA and RNA extractions were performed.

### Isolation of DNA and Total RNA from Human Liver Tissues

Tissues in RNAlater were thawed at room temperature, washed with DEPC-treated water and homogenized in 1 ml Trizol (Invitrogen) using a Pro200 homogenizer (Pro Scientific Inc.). Both DNA and total RNA were extracted according to the manufacturer's protocol. Extracted DNA and total RNA were quantitated using a Biophotometer (Eppendorf). Total RNA was purified by DNase I (Takara) treatment followed by phenol/chloroform extraction and ethanol precipitation. Purified RNA was dissolved in DEPC-treated water, quantitated, diluted to approximately 1 µg/µl and stored at −70°C.

### Absolute Quantitation of mRNA level by Real-time PCR

All primers for real-time quantitative PCR were designed to specifically amplify fragments of 80–180 bp in length inside single exons using the online program Primer3 (http://frodo.wi.mit.edu/primer3/input.htm). The sequence for each primer is listed in Table S3. Reverse transcription reactions were prepared in a total volume of 20 µl containing 1× RT buffer, 0.5 mM dNTP, 0.5 µM poly24dT, 5 µM N9, 20 U RNase inhibitor (BIO BASIC INC.) and 100 U M-MLV(H-) reverse transcriptase (Promega). The reactions were incubated at room temperature for 10 min, 42°C for 60 min and 70°C for 15 min to inactivate the enzyme. The reverse transcription (RT) product for each sample was used to create three dilutions: 4-fold, 20-fold and 40000-fold. Human genomic DNA from Promega (Catalog No. G152A) was serially diluted as the input DNA for the construction of standard curves as described in Jiang et al. [33]. The copy number of each gene in the input standard genomic DNA was calculated by multiplying the DNA amount and N_0_ (the copy number of the corresponding gene per nanogram human genomic DNA). N_0_ was 280 for all *P450s* and transcription factor genes assuming that there were only 2 copies in each cell (Qiagen Genomic DNA Handbook). For the three reference genes, N_0_ was 1660 for *GAPDH*, 4200 for *ACTB* and 22400 for *18*S *rRNA*. These three numbers were estimated by comparing standard curves of these reference genes and those of genes with 2 copies per genome (data not shown). The cDNA molecule numbers of the 14 target genes and 3 reference genes (*18S rRNA*, *GAPDH* and *ACTB*) in the RT products were determined using the absolute quantification program of the SDS2.0 software on an ABI7900 machine (Applied Biosystems). The expression levels of the 14 target genes and 2 reference genes (*GAPDH* and *ACTB*) were normalized to *18S rRNA* by dividing the cDNA molecule number of each target gene by that of *18S rRNA* and multiplying by 10^7^. Each real time PCR reaction included 1× QuantiTect SYBR Green PCR Master Mix (Qiagen Inc.), 0.3 µM each primer and 2 µl of diluted RT product (40000-fold dilution for *18S rRNA* and 20-fold dilution for other targets) or serially diluted standard genomic DNA. The PCR program was 95°C for 15 min followed by 40 cycles of 94°C for 15 s, 57°C for 30 s and 72°C for 30 s.

### Quantification of CYP2D6 copy number

Two primer pairs specific for *CYP2D6* were designed to amplify different fragments, and one primer pair was designed for ribonuclease P RNA component H1 (*RPPH1*). The primer sequences and amplicon sizes are listed in Table S3. The molecule quantity of *CYP2D6* and *RPPH1* genes in 2 µl DNA samples were separately determined as described above. The copy number of *CYP2D6* in the control DNA from Promega (Cat. No. G152A) was estimated to be 2 by comparing the standard curve of *CYP2D6* with that of *RPPH1* (data not shown). Two measurements obtained using different primer pairs were averaged to obtain the quantity of *CYP2D6* molecules. The *CYP2D6* copy number was estimated by rounding the ratio of the quantity of *CYP2D6* molecules to that of *RPPH1*.

### SNP Genotyping

In total, 15 SNPs in 9 genes were selected and typed in 96 DNA samples extracted from liver tissue samples using the Multiplex SNaPshot kit (Applied Biosystems Inc.) (Table S2). For the *CYP1A1*, *CYP1A2*, *CYP2C9*, *CYP2C19* and *CYP3A4* genes, two SNPs within the gene with low linkage disequilibrium (LD) (r^2^<0.5) and high minor allele frequency (MAF) (>20%) were selected. LD and MAF were calculated from genotyping data released by the HapMap project (http://www.hapmap.org). These ten SNPs were also typed in heterozygous cDNA samples as markers to determine allelic expression ratios. Rs776746 in *CYP3A5* contains the CYP3A5*3 allele, which causes improper splicing and results in a nonfunctional, truncated protein. The selected SNPs in *HNF1A* and *AHR* are non-synonymous (rs1169288, rs2464196 and rs2066853). Three multiple PCR and extension panels were designed for the SNaPshot reactions. A 10 µl mixture was prepared for each multiple PCR reaction and included 1× HotStarTaq buffer, 2.8 mM Mg^2+^, 0.2 mM dNTP, 0.1–0.3 µM primers, 0.3 U HotStarTaq polymerase (Qiagen Inc.) and 1 µl template DNA. The cycling program was 95°C for 15 min; 11 cycles of 94°C for 20 s, 62°C −0.5°C/cycle for 40 s and 72°C for 80 s; 25 cycles of 94°C for 20 s, 56°C for 30 s and 72°C for 80 s and 72°C for 5 min. The PCR products were purified with the Exo I and SAP enzymes, and subsequent SNaPshot extension was carried out as described in the manufacturer's protocol. Extension products were separated by gel electrophoresis and analyzed using the ABI3730XL (Applied Biosystems) system. Data collected on the ABI3730XL system were analyzed with GeneMapper 4.0.

### Measurement of allelic expression ratios

**Figure 6:**
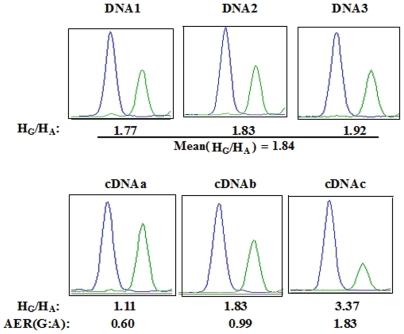
Illustration of the method used to measure allelic expression ratios in three cDNA samples using SNaPshot single nucleotide extension. For each marker SNP, two extension products were produced with two different fluorescent labels, corresponding to two different alleles (blue for the G allele and green for the A allele). Extension products were separated by capillary electrophoresis on an ABI sequencer, and data were analyzed with GeneMapper 4.0. HG/HA denotes the ratio of the peak height of the G allele to that of the A allele. The HG/HA ratio was first calculated for three DNA heterozygotes (DNA1, DNA2 and DNA3) and three cDNA heterozygotes (cDNAa, cDNAb and cDNAc). The HG/HA ratios for the three DNA samples (1.77, 1.83 and 1.92, respectively) were averaged to obtain a mean HG/HA of 1.84, corresponding to an allelic ratio of 1∶1. The allelic expression ratios - designated here as AER (G: A) - in these three cDNA samples were determined to be 0.60, 0.99 and 1.83, respectively, by dividing the HG/HA ratios (1.11, 1.83 and 3.37, respectively) by 1.84.

All heterozygous cDNA samples and 5 heterozygous DNA samples for each marker SNP in the *CYP1A1*, *CYP1A2*, *CYP2C9*, *CYP2C19* and *CYP3A4* genes were subjected to single SNaPshot reactions. Four-fold dilutions of the cDNA samples were used for amplification of fragments containing intronic SNPs, and twenty-fold dilutions were used for exonic SNPs. The whole procedure including PCR amplification, SNaPshot extension, electrophoretic separation and data analysis was similar to that described in the above paragraph, except that only 2.0 mM Mg^2+^ and one pair of primers were included in the PCR reaction, and a single extension primer was added in the SNaPshot extension reaction. Data collected on the ABI3730XL were analyzed with GeneMapper 4.0, and the peak height for each allele was exported for further analysis. The allelic expression ratio was determined by the peak height ratio of two alleles in the cDNA samples divided by the average peak height ratio in five DNA samples that were assumed to have a 1∶1 ratio of the two alleles. The basic rationale for measurement of allelic expression ratio is illustrated in Fig. 6.

The allelic expression imbalance (AEI) assay was carried out by detecting the expression ratio of two alleles in heterozygous cDNA samples in which the expression level of the two alleles were controlled by each other. Fig. 6 illustrates the basic concept of this assay using the single fluorescent-labeled nucleotide extension method (SNaPshot, ABI). Theoretically, this assay can significantly reduce the noise that results from variations in activity of regulatory factors and improve the sensitivity of detection.

### Statistical analysis

SPSS VERSION 15.0 (SPSS Inc., Chicago, IL) was used for statistical analyses and for BOXPLOT construction. Both the Kruskal-Wallis test and ANOVA were performed to examine the associations between genotype and mRNA content. Pearson's or Spearman's rho correlation coefficients between mRNA levels or between the copy number and mRNA level of *CYP2D6* were identified using bivariate regression analysis. Multivariate logistic regression was used to adjust for age and smoking history. The UPGMA tree for seven *P450s* and seven regulator genes was constructed using the MEGA4 program based on the pairwise Spearman's rho correlation coefficient data.

## Supporting Information

Table S1 Abbreviation of gene names.
(DOC)

Table S2 Characterization of SNP information and primer sequence for multiplex SNaPshot reactions.
(DOC)

Table S3 Primers for quantitative real time PCR.
(DOC)

Table S4 Inter-individual variation in the mRNA level of housekeeping, *P450* and regulatory genes (Normalized to *18SrRNA*).
(DOC)

Table S5 Correlations among the mRNA levels of two housekeeping genes, seven *P450* genes and seven regulator genes.
(DOC)

Table S6 Partial correlations among *P450* genes and regulator genes by controlling on *GAPDH* and *ACTB* in mRNA level.
(DOC)
