# Supplementary material for: Functional Evaluation of Genetic and Environmental Regulators of P450 mRNA Levels
Source: PLoS One. 2011 Oct 5;6(10):e24900. doi: 10.1371/journal.pone.0024900 (PMC3187744; doi:10.1371/journal.pone.0024900)
Supplement: Table S1 — Abbreviation of gene names. (DOC) [file pone.0024900.s001.doc]

**Table S1** Abbreviation of gene names

| Gene Name | Abbreviation |
| --- | --- |
| 18S ribosomal RNA | *18SrRNA* |
| Glyceraldehyde-3-phosphate dehydrogenase | *GAPDH* |
| beta-actin | *ACTB* |
| Cytochrome P450 1A1 | *CYP1A1* |
| Cytochrome P450 1A2 | *CYP1A2* |
| Cytochrome P450 2C9 | *CYP2C9* |
| Cytochrome P450 2C19 | *CYP2C19* |
| Cytochrome P450 2D6 | *CYP2D6* |
| Cytochrome P450 3A4 | *CYP3A4* |
| Cytochrome P450 3A5 | *CYP3A5* |
| Upstream transcription factor 1 | *USF1* |
| Constitutive androstane receptor | *CAR* |
| Pregnane X receptor | *PXR* |
| Hepatocyte nuclear factor 4, alpha | *HNF4A* |
| Hepatocyte nuclear factor 1, alpha | *HNF1A* |
| Aryl hydrocarbon receptor | *AHR* |
| Aryl hydrocarbon receptor nuclear translocator | *ARNT* |
