# Supplementary material for: Functional Evaluation of Genetic and Environmental Regulators of P450 mRNA Levels
Source: PLoS One. 2011 Oct 5;6(10):e24900. doi: 10.1371/journal.pone.0024900 (PMC3187744; doi:10.1371/journal.pone.0024900)
Supplement: Table S2 — Characterization of SNP information and primer sequence for multiplex SNaPshot reactions. (DOC) [file pone.0024900.s002.doc]

**Table S2** Characterization of SNP information and primer sequence for multiplex SNaPshot reactions

| SNP loci | Gene | SNP property | PCR Primer(5' 3') | Fragment Size | PCR Panel | Extension Primer(5' 3') |
| --- | --- | --- | --- | --- | --- | --- |
| rs1048943 | *CYP1A1* | Ile462>Val462 | CAGCCCAGATATGCAAAACTGC | 383bp | 1 | CCCCCCCCCCCAAGACCTCCCAGCGGGCAA |
|  |  |  | TCTCACCCCTGATGGTGCTAT |  |  |  |
| rs4646421 | *CYP1A1* | Intron1 | AGGTGTATCACCCCGAGTCCT | 413bp | 2 | AATTTCCAGCTGCTGTCACAT |
|  |  |  | CCAGCTGCCTTTAAACTGTGG |  |  |  |
| rs17861162 | *CYP1A2* | 3'UTR | ACTACAGGCACACACCACCA | 249bp | 3 | CCCCCCCCCCCCCCCCCCCCCCCCTACAGGTGTGAGCCACGGTG |
|  |  |  | CAGGCTAGGGGGAAATGTG |  |  |  |
| rs762551 | *CYP1A2* | Intron1 | GAGAGAGCCAGCGTTCATGTT | 269bp | 2 | CCCCCCCCCCCCCCCCCCCCCCCAAGGGTGAGCTCTGTGGGC |
|  |  |  | ACAGACTGGGACAATGCCATC |  |  |  |
| rs1934967 | *CYP2C9* | Intron7 | TGGCAGTGTGTGTTCTCCAG | 307bp | 3 | CCCTATTAGTTTGTCAATTTCCCAAAAC |
|  |  |  | TATCTAGGGAAAACCAAAGACTCGA |  |  |  |
| rs1934969 | *CYP2C9* | Intron8 | ACAGGGTGCCTGTTAAGATCTGATAT | 541bp | 3 | CCCCCCCCCCCCCCCCAGAATATGCAATAACTGTTCAGTG |
|  |  |  | TTACAGATAGTGAAAGATGGATAATGCC |  |  |  |
| rs4388808 | *CYP2C19* | Intron3 | ATGGCCCTATCACACCCCTAG | 249bp | 1 | CCATTATTAGTAATAACTGTAATTTTTATTCCTTAAGT |
|  |  |  | ACTTCATAGGTGAGTAGAGTTGGCAG |  |  |  |
| rs4244285 | *CYP2C19* | Pro227>Pro227 | CAACCAGAGCTTGGCATATTGTATC | 318bp | 3 | CCCCCCCCCTTTTTAAGTAATTTGTTATGGGTTCC |
|  |  |  | CAAAACTAGTCAATGAATCACAAATACG |  |  |  |
| rs2267447 | *CYP2D6* | Intron4 | GAGCGAGAGACCGAGGAGTC | 139bp | 3 | CCCCTCGGAAGAGCAGGATTTGC |
|  |  |  | CCAGGCCCTTCTTACAGTGG |  |  |  |
| rs2246709 | *CYP3A4* | Intron7 | TCACAAACCCTGTCATCATATGC | 308bp | 2 | CCCCGCAACCACTAATCAACTTTCTGC |
|  |  |  | AATCTCATGGGATTTAGCAAAGG |  |  |  |
| rs33972239 | *CYP3A4* | 3'UTR | TGGTCATTGTAATCACTGTTGGC | 299bp | 1 | CCCCCCCCCCCCCCCCCCCCCCCCCAAGCCTGGCCTACATGGT |
|  |  |  | ATTACAGGCGAGTCCACCAT |  |  |  |
| rs776746 | *CYP3A5* | Intron3 | CGTATGTACCACCCAGCTTAACG | 339bp | 1 | CCCCCCTCTTTAAAGAGCTCTTTTGTCTTTCA |
|  |  |  | CAGGGAGTTGACCTTCATACGTT |  |  |  |
| rs1169288 | *HNF1A* | Ile27>Leu27 | GAGTCAGGGCTGAGCAAAGAG | 220bp | 2 | CCCCCCCCCCCCCCCCCCCCCCCCCCCCCCGCTGAGCAAAGAGGCACTG |
|  |  |  | TCTCCAGCTCTTTGAGGATGG |  |  |  |
| rs2464196 | *HNF1A* | Ser487>Asn487 | TCAGAGAGGGGGAATGACTTG | 422bp | 1 | CAGAGCCATGTGACCCAGA |
|  |  |  | TCATCATCTCCTGCTGTGTGG |  |  |  |
| rs2066853 | *AHR* | Arg554>Lys554 | ATACTGCACCGATGGGAAATG | 388bp | 2 | CCCCCCCCCCCCCAAAAATTTTTCATTCTGCATGTGT |
|  |  |  | TTTTGGTGATGTTGCTGTTGC |  |  |  |
