# Supplementary material for: Functional Evaluation of Genetic and Environmental Regulators of P450 mRNA Levels
Source: PLoS One. 2011 Oct 5;6(10):e24900. doi: 10.1371/journal.pone.0024900 (PMC3187744; doi:10.1371/journal.pone.0024900)
Supplement: Table S3 — Primers for quantitative real time PCR. (DOC) [file pone.0024900.s003.doc]

**Table S3** Primers for quantitative real time PCR

| Gene | Forward Primer (5´→3´) | Reverse Primer (5´→3´) | Fragment Size(bp) |
| --- | --- | --- | --- |
| *18SrRNA* | AGAAACGGCTACCACATCCAA | CAATTACAGGGCCTCGAAAGA | 114 |
| *GAPDH* | AACAGGGTGGTGGACCTCAT | GGAGGGGAGATTCAGTGTGG | 153 |
| *ACTB* | GTGACAGCAGTCGGTTGGAG | AGGACTGGGCCATTCTCCTT | 176 |
| *CYP1A1* | CCACAGCCCAGATAGCAAAAC | AGGCTGGCCTATGTGGTCTAA | 80 |
| *CYP1A2* | GCCATTAACAAGCCCTTGAG | ATGGCCAGGAAGAGGAAGAT | 107 |
| *CYP2C9* | CTCTCTTTCCTCTGGGGCATT | GGAAACTCTCCGTAATGGAGGTC | 124 |
| *CYP2C19* | CGGATTTGTGTGGGAGAGGG | GCAAATCCATTGACAACAGGAGTT | 130 |
| *CYP2D6* | ATAGTGGTGGCTGACCTGTTCTC | CCCAGGCCAGCGTGGTCG | 59 |
| *CYP3A4* | GCTCTTCAAGAAATCTGTGCCTG | TCTACACAGACAATGAGAGAGCTCAA | 160 |
| *CYP3A5* | CCCCTTGAAATTAGACACGCA | CACAGCTTTCTTGAAGACCAAAGT | 136 |
| *USF1* | ACCACGGATTAGAGGTCGTCA | AAGTGGGGCAGTGAAGGAAAG | 133 |
| *CAR* | GGAGCAGCTGTGGAAATCTGT | GGCTCCATCTTCAATTGTGTAGC | 105 |
| *PXR* | ACAGCTGGCTAGCATTCCTCA | CTTGCCTCTCTGATGGTCCTG | 131 |
| *HNF4A* | AGCGATCCAGGGAAGATCAAG | AGCAGCAGCAGCTCTCCAA | 113 |
| *HNF1A* | GTCGTGGAGAGCTAGGAGCAA | GCCCCCTTCAGTTCCAAGTAA | 99 |
| *AHR* | CCCAATTTTGACCCTGGTTTT | ATGCCTCCATGTGAACTTGCT | 100 |
| *ARNT* | CCCTGCTCTGTTGCCTCTCTA | TTCACCCAGCCTCAAATTTTC | 96 |
| *CYP2D6^1^* | AGGTGGATGCACAAAGAGTGG | ACCCACTCCAACCCTATGCTC | 89 |
| *CYP2D6^2^* | CATGAACTTTGCTGGGACACC | GCCTCCTATGTTGGAGGAGGTC | 84 |
| *RPPH1^3^* | GGCGGAGGAGAGTAGTCTGAAT | GGCCAGCGAAGTGAGTTCAA | 82 |

Note: ^1, 2, 3^Primers for quantitative PCR to determine the number of CYP2D6 copies.
