# Supplementary material for: Functional Evaluation of Genetic and Environmental Regulators of P450 mRNA Levels
Source: PLoS One. 2011 Oct 5;6(10):e24900. doi: 10.1371/journal.pone.0024900 (PMC3187744; doi:10.1371/journal.pone.0024900)
Supplement: Table S4 — Inter-individual variation in the mRNA level of housekeeping, P450 and regulatory genes (Normalized to 18SrRNA). (DOC) [file pone.0024900.s004.doc]

**Table S**4 Interindividual variation in the mRNA level of housekeeping, P450 and regulatory genes (Normalized to 18SrRNA)

|  | Median | Mean | STDEV^1^ | CV^2^ | Minimal M.N.^3^ | Maximal M.N.^3^ | Ratio (Max/Min)^4^ | Mean (Bottom 5%)^5^ | Mean (Top 5%)^6^ | Ratio (T5/B5)^7^ |
| --- | --- | --- | --- | --- | --- | --- | --- | --- | --- | --- |
| *GAPDH* | 9431 | 10449 | 4992 | 0.48 | 3251 | 27639 | 8.5 | 3873 | 23897 | 6.2 |
| *ACTB* | 9211 | 10523 | 5342 | 0.51 | 3270 | 34497 | 10.5 | 3914 | 25831 | 6.6 |
| *CYP1A1* | 316 | 522 | 598 | 1.15 | 22 | 3039 | 138.1 | 38 | 2571 | 67.7 |
| *CYP1A2* | 2843 | 3381 | 2064 | 0.61 | 785 | 10947 | 13.9 | 880 | 9074 | 10.3 |
| *CYP2C9* | 6300 | 7056 | 3463 | 0.49 | 2309 | 16677 | 7.2 | 2764 | 15730 | 5.7 |
| *CYP2C19* | 1068 | 1299 | 869 | 0.67 | 108 | 4829 | 44.7 | 267 | 3975 | 14.9 |
| *CYP2D6* | 4335 | 5215 | 4022 | 0.77 | 244^8^ | 16615 | 68.1 | 374 | 15400 | 41.2 |
| *CYP3A4* | 7445 | 9525 | 7348 | 0.77 | 874 | 55584 | 63.6 | 2120 | 32715 | 15.4 |
| *CYP3A5* | 1984 | 2445 | 1673 | 0.68 | 723 | 10332 | 14.3 | 818 | 7732 | 9.5 |
| *USF1* | 83 | 87 | 19 | 0.22 | 51 | 137 | 2.7 | 53 | 132 | 2.5 |
| *CAR* | 684 | 718 | 289 | 0.40 | 284 | 1777 | 6.3 | 314 | 1482 | 4.7 |
| *PXR* | 569 | 577 | 229 | 0.40 | 233 | 1334 | 5.7 | 248 | 1129 | 4.5 |
| *HNF4A* | 980 | 998 | 315 | 0.32 | 358 | 1984 | 5.5 | 487 | 1776 | 3.6 |
| *HNF1A* | 146 | 152 | 50 | 0.33 | 61 | 299 | 4.9 | 74 | 281 | 3.8 |
| *AHR* | 342 | 399 | 170 | 0.43 | 144 | 957 | 6.6 | 161 | 836 | 5.2 |
| *ARNT* | 206 | 214 | 77 | 0.36 | 62 | 445 | 7.2 | 88 | 386 | 4.4 |

Note: 1: standard deviation; 2: Coefficient of Variation; 3: molecule number; 4: the ratio of maximal M.N. to minimal M.N.; 5: the mean of the bottom 5 M.N.s; 6: the mean of the top 5 M.N.s; 7: the ratio of Mean (TOP 5%) to Mean (Bottom 5%); 8: Samples homozygote for CYP2D6 deletion were excluded.
