# Supplementary material for: Functional Evaluation of Genetic and Environmental Regulators of P450 mRNA Levels
Source: PLoS One. 2011 Oct 5;6(10):e24900. doi: 10.1371/journal.pone.0024900 (PMC3187744; doi:10.1371/journal.pone.0024900)
Supplement: Table S5 — Correlations among the mRNA levels of two housekeeping genes, seven P450 genes and seven regulator genes. (DOC) [file pone.0024900.s005.doc]

**Table S**5 Correlations among the mRNA levels of two housekeeping genes, seven P450 genes and seven regulator genes

|  |  | *GAPDH* | *ACTB* | *CYP1A1* | *CYP1A2* | *CYP2C9* | *CYP2C19* | *CYP2D6* | *CYP3A4* | *CYP3A5* | *USF1* | *CAR* | *PXR* | *HNF4A* | *HNF1A* | *AHR* |
| --- | --- | --- | --- | --- | --- | --- | --- | --- | --- | --- | --- | --- | --- | --- | --- | --- |
| *ARNT* | ρ | 0.828 | 0.763 | 0.503 | 0.617 | 0.881 | 0.559 | 0.363 | 0.705 | 0.595 | 0.631 | 0.885 | 0.849 | 0.809 | 0.875 | 0.776 |
|  | Sig. | 0.000 | 0.000 | 0.000 | 0.000 | 0.000 | 0.000 | 0.000 | 0.000 | 0.000 | 0.000 | 0.000 | 0.000 | 0.000 | 0.000 | 0.000 |
| *AHR* | ρ | 0.726 | 0.757 | 0.563 | 0.638 | 0.768 | 0.447 | 0.221 | 0.618 | 0.559 | 0.646 | 0.761 | 0.668 | 0.600 | 0.747 |  |
|  | Sig. | 0.000 | 0.000 | 0.000 | 0.000 | 0.000 | 0.000 | 0.000 | 0.000 | 0.000 | 0.000 | 0.000 | 0.000 | 0.000 | 0.000 |  |
| *HNF1A* | ρ | 0.839 | 0.818 | 0.520 | 0.619 | 0.879 | 0.510 | 0.304 | 0.614 | 0.554 | 0.685 | 0.863 | 0.871 | 0.763 |  |  |
|  | Sig. | 0.000 | 0.000 | 0.000 | 0.000 | 0.000 | 0.000 | 0.031 | 0.000 | 0.000 | 0.000 | 0.000 | 0.000 | 0.000 |  |  |
| *HNF4A* | ρ | 0.713 | 0.666 | 0.402 | 0.509 | 0.740 | 0.395 | 0.344 | 0.493 | 0.515 | 0.552 | 0.769 | 0.714 |  |  |  |
|  | Sig. | 0.000 | 0.000 | 0.000 | 0.000 | 0.000 | 0.000 | 0.003 | 0.000 | 0.000 | 0.000 | 0.000 | 0.000 |  |  |  |
| *PXR* | ρ | 0.849 | 0.745 | 0.426 | 0.609 | 0.880 | 0.575 | 0.333 | 0.681 | 0.553 | 0.594 | 0.856 |  |  |  |  |
|  | Sig. | 0.000 | 0.000 | 0.000 | 0.000 | 0.000 | 0.000 | 0.001 | 0.000 | 0.000 | 0.000 | 0.000 |  |  |  |  |
| *CAR* | ρ | 0.835 | 0.732 | 0.478 | 0.607 | 0.876 | 0.537 | 0.398 | 0.692 | 0.578 | 0.598 |  |  |  |  |  |
|  | Sig. | 0.000 | 0.000 | 0.000 | 0.000 | 0.000 | 0.000 | 0.000 | 0.000 | 0.000 | 0.000 |  |  |  |  |  |
| *USF1* | ρ | 0.578 | 0.674 | 0.476 | 0.511 | 0.627 | 0.347 | 0.245 | 0.417 | 0.398 |  |  |  |  |  |  |
|  | Sig. | 0.000 | 0.000 | 0.000 | 0.000 | 0.000 | 0.001 | 0.017 | 0.000 | 0.000 |  |  |  |  |  |  |
| *CYP3A5* | ρ | 0.614 | 0.569 | 0.252 | 0.407 | 0.645 | 0.413 | 0.082 | 0.517 |  |  |  |  |  |  |  |
|  | Sig. | 0.000 | 0.000 | 0.014 | 0.000 | 0.000 | 0.000 | N.S. | 0.000 |  |  |  |  |  |  |  |
| *CYP3A4* | ρ | 0.652 | 0.552 | 0.500 | 0.551 | 0.778 | 0.558 | 0.303 |  |  |  |  |  |  |  |  |
|  | Sig. | 0.000 | 0.000 | 0.000 | 0.000 | 0.000 | 0.000 |  |  |  |  |  |  |  |  |  |
| *CYP2D6* | ρ | 0.246 | 0.275 | 0.192 | 0.252 | 0.312 | 0.195 |  |  |  |  |  |  |  |  |  |
|  | Sig. | 0.016 | 0.007 | N.S. | 0.014 | 0.002 | N.S |  |  |  |  |  |  |  |  |  |
| *CYP2C19* | ρ | 0.496 | 0.419 | 0.361 | 0.514 | 0.575 |  |  |  |  |  |  |  |  |  |  |
|  | Sig. | 0.000 | 0.000 | 0.000 | 0.000 | 0.000 |  |  |  |  |  |  |  |  |  |  |
| *CYP2C9* | ρ | 0.868 | 0.828 | 0.536 | 0.651 |  |  |  |  |  |  |  |  |  |  |  |
|  | Sig. | 0.000 | 0.000 | 0.000 | 0.000 |  |  |  |  |  |  |  |  |  |  |  |
| *CYP1A2* | ρ | 0.630 | 0.668 | 0.875 |  |  |  |  |  |  |  |  |  |  |  |  |
|  | Sig. | 0.000 | 0.000 | 0.000 |  |  |  |  |  |  |  |  |  |  |  |  |
| *CYP1A1* | ρ | 0.474 | 0.564 |  |  |  |  |  |  |  |  |  |  |  |  |  |
|  | Sig. | 0.000 | 0.000 |  |  |  |  |  |  |  |  |  |  |  |  |  |
| *ACTB* | ρ | 0.848 |  |  |  |  |  |  |  |  |  |  |  |  |  |  |
|  | Sig. | 0.000 |  |  |  |  |  |  |  |  |  |  |  |  |  |  |

ρ, Spearman’s rho correlation coefficient; Sig, significance (p value); N.S., not significant (p>0.05), Values of 0 indicate that p<0.0005
