# Supplementary material for: Functional Evaluation of Genetic and Environmental Regulators of P450 mRNA Levels
Source: PLoS One. 2011 Oct 5;6(10):e24900. doi: 10.1371/journal.pone.0024900 (PMC3187744; doi:10.1371/journal.pone.0024900)
Supplement: Table S6 — Partial correlations among P450 genes and regulator genes by controlling on GAPDH and ACTB in mRNA level. (DOC) [file pone.0024900.s006.doc]

**Table S6 Partial correlations among P450 genes and regulator genes by controlling on GAPDH and ACTB** in mRNA level

|  |  | *CYP1A1* | *CYP1A2* | *CYP2C9* | *CYP2C19* | *CYP2D6* | *CYP3A4* | *CYP3A5* | *USF1* | *CAR* | *PXR* | *HNF4A* | *HNF1A* | *AHR* |
| --- | --- | --- | --- | --- | --- | --- | --- | --- | --- | --- | --- | --- | --- | --- |
| *ARNT* | ρ | 0.052 | 0.112 | 0.574 | 0.4 | 0.241 | 0.432 | 0.122 | 0.323 | 0.58 | 0.548 | 0.544 | 0.628 | 0.397 |
|  | Sig. | N.S. | N.S. | 0.000 | 0.000 | 0.020 | 0.000 | N.S. | 0.002 | 0.000 | 0.000 | 0.000 | 0.000 | 0.000 |
| *AHR* | ρ | 0.142 | 0.232 | 0.364 | 0.208 | -0.011 | 0.239 | 0.109 | 0.2 | 0.475 | 0.197 | 0.106 | 0.381 |  |
|  | Sig. | N.S. | 0.025 | 0.000 | 0.046 | N.S. | 0.021 | N.S. | N.S. | 0.000 | N.S. | N.S. | 0.000 |  |
| *HNF1A* | ρ | 0.036 | 0.111 | 0.504 | 0.262 | 0.162 | 0.272 | -0.047 | 0.44 | 0.666 | 0.66 | 0.545 |  |  |
|  | Sig. | N.S. | N.S. | 0.000 | 0.011 | N.S. | 0.008 | N.S. | 0.000 | 0.000 | 0.000 | 0.000 |  |  |
| *HNF4A* | ρ | 0.037 | 0.135 | 0.374 | 0.103 | 0.29 | 0.113 | -0.088 | 0.353 | 0.487 | 0.44 |  |  |  |
|  | Sig. | N.S. | N.S. | 0.000 | N.S. | 0.005 | N.S. | N.S. | 0.001 | 0.000 | 0.000 |  |  |  |
| *PXR* | ρ | 0.042 | 0.127 | 0.571 | 0.338 | 0.363 | 0.316 | -0.095 | 0.282 | 0.619 |  |  |  |  |
|  | Sig. | N.S. | N.S. | 0.000 | 0.001 | 0.000 | 0.002 | N.S. | 0.006 | 0.000 |  |  |  |  |
| *CAR* | ρ | 0.123 | 0.235 | 0.659 | 0.284 | 0.323 | 0.382 | 0.067 | 0.3 |  |  |  |  |  |
|  | Sig. | N.S. | 0.024 | 0.000 | 0.006 | 0.002 | 0.000 | N.S. | 0.004 |  |  |  |  |  |
| *USF1* | ρ | 0.217 | 0.268 | 0.236 | 0.098 | 0.107 | -0.006 | -0.129 |  |  |  |  |  |  |
|  | Sig. | 0.037 | 0.009 | 0.023 | N.S. | N.S. | N.S. | N.S. |  |  |  |  |  |  |
| *CYP3A5* | ρ | -0.346 | -0.23 | 0.261 | -0.02 | -0.139 | 0.203 |  |  |  |  |  |  |  |
|  | Sig. | 0.001 | 0.027 | 0.012 | N.S. | N.S. | N.S. |  |  |  |  |  |  |  |
| *CYP3A4* | ρ | 0.041 | 0.047 | 0.663 | 0.602 | 0.116 |  |  |  |  |  |  |  |  |
|  | Sig. | N.S. | N.S. | 0.000 | 0.000 | N.S. |  |  |  |  |  |  |  |  |
| *CYP2D6* | ρ | 0.088 | 0.083 | 0.13 | 0.06 |  |  |  |  |  |  |  |  |  |
|  | Sig. | N.S. | N.S. | N.S. | N.S. |  |  |  |  |  |  |  |  |  |
| *CYP2C19* | ρ | 0.21 | 0.276 | 0.445 |  |  |  |  |  |  |  |  |  |  |
|  | Sig. | 0.044 | 0.008 | 0.000 |  |  |  |  |  |  |  |  |  |  |
| *CYP2C9* | ρ | 0.046 | 0.188 |  |  |  |  |  |  |  |  |  |  |  |
|  | Sig. | N.S. | N.S. |  |  |  |  |  |  |  |  |  |  |  |
| *CYP1A2* | ρ | 0.807 |  |  |  |  |  |  |  |  |  |  |  |  |
|  | Sig. | 0.000 |  |  |  |  |  |  |  |  |  |  |  |  |

ρ, Spearman’s rho correlation coefficient; Sig, significance(p value); N.S., not significant (p>0.05), Values of 0 indicate that p<0.0005
